# Assessment of Sodium Knowledge and Urinary Sodium Excretion among Regions of the United Arab Emirates: A Cross-Sectional Study

**DOI:** 10.3390/nu12092747

**Published:** 2020-09-09

**Authors:** Amjad H. Jarrar, Lily Stojanovska, Vasso Apostolopoulos, Leila Cheikh Ismail, Jack Feehan, Eric O. Ohuma, Ala Z. Ahmad, Asma A. Alnoaimi, Latifa S. Al Khaili, Najah H. Allowch, Fatima T. Al Meqbaali, Usama Souka, Ayesha S. Al Dhaheri

**Affiliations:** 1 Food, Nutrition and Health Department, College of Food and Agriculture, United Arab Emirates University, Al Ain 15551, UAE; AmjadJ@uaeu.ac.ae (A.H.J.); lily.stojanovska@uaeu.ac.ae (L.S.); 201050547@uaeu.ac.ae (A.Z.A.); 201050511@uaeu.ac.ae (A.A.A.); 201005105@uaeu.ac.ae (L.S.A.K.); 201050006@uaeu.ac.ae (N.H.A.); fatmadiab@uaeu.ac.ae (F.T.A.M.); usamasouka@uaeu.ac.ae (U.S.); 2 Institute for Health and Sport, Victoria University, Melbourne 14428, Australia; vasso.apostolopoulos@vu.edu.au (V.A.); jfeehan@student.unimelb.edu.au (J.F.); 3 Clinical Nutrition and Dietetics Department, College of Health Sciences, University of Sharjah, Sharjah 27272, UAE; lcheikhismail@sharjah.ac.ae; 4 Nuffield Department of Women’s & Reproductive Health, University of Oxford, Oxford OX1 2JD, UK; 5 Department of Medicine—Western Health, The University of Melbourne, Melbourne 3021, Australia; 6 Maternal, Adolescent, Reproductive & Child Health (MARCH) Centre, London School of Hygiene &Tropical Medicine (LSHTM), London WC1E 7HT, UK; eric.ohuma@ndm.ox.ac.uk; 7 Centre for Tropical Medicine and Global Health, Nuffield Department of Medicine, University of Oxford, Oxford OX3 7LG, UK

**Keywords:** urinary sodium excretion, urinary potassium excretion, salt, sodium, non-communicable diseases, United Arab Emirates

## Abstract

Non-communicable diseases (NCDs) such as cardiovascular disease, cancer and diabetes, are increasing worldwide and cause 65% to 78% of deaths in the Gulf Cooperation Council (GCC). A random sample of 477 healthy adults were recruited in the United Arab Emirates (UAE) in the period March–June 2015. Demographic, lifestyle, medical, anthropometric and sodium excretion data were collected. A questionnaire was used to measure knowledge, attitude and practice regarding salt. Mean sodium and potassium excretion were 2713.4 ± 713 mg/day and 1803 ± 618 mg/day, respectively, significantly higher than the World Health Organization (WHO) recommendations for sodium (2300 mg/day) and lower for potassium (3150 mg/day). Two-thirds (67.4%) exceeded sodium guidelines, with males 2.6 times more likely to consume excessively. The majority of the participants add salt during cooking (82.5%) and whilst eating (66%), and 75% identified processed food as high source of salt. Most (69.1%) were aware that excessive salt could cause disease. Most of the UAE population consumes excess sodium and insufficient potassium, likely increasing the risk of NCDs. Despite most participants being aware that high salt intake is associated with adverse health outcomes, this did not translate into salt reduction action. Low-sodium, high-potassium dietary interventions such as the Mediterranean diet are vital in reducing the impact of NCDs in the UAE.

## 1. Introduction

Chronic diseases are long-lasting conditions with continuous effects, and include cardiovascular disease (CVD), chronic respiratory disease (CRD), cancer and type 2 diabetes (T2D). Globally, their incidence is increasing becoming a growing burden to global economies and people’s quality of life. Collectively, these non-communicable diseases (NCDs) are the leading cause of death globally [1], making it a significant priority in healthcare systems. In some countries, up to 40% of those dying from NCDs are younger than 60 years of age [1].

The rapid improvement in the socio-economic status of the countries making up the Gulf Cooperation Council (GCC) (Bahrain, Kuwait, Oman, Qatar, Saudi Arabia and the United Arab Emirates (UAE) has contributed to changing food consumption patterns, lifestyle habits and health status over the last four decades, with diet quality decreasing through the addition of more processed Westernized food. These changes have resulted in an increasingly sedentary lifestyle, high blood pressure and obesity, known to be major risk factors of NCDs [2,3]. Thus, it is not surprising that CVD is the major cause of morbidity and mortality in the Gulf region [4]. It is estimated that NCDs cause between 65% and 78% of deaths in the GCC member countries [5].

In particular, the lifestyle of the Emirati population has changed considerably in the last 40 years due to rapid improvement in socio-economic indicators in the UAE. This transition has led to less physical activity and altered eating habits, including increased intake of processed foods. These changes, in addition to the adoption of a Western lifestyle and diet, have led to the rise in prevalence of overweight, obesity and the risk of metabolic syndrome in the UAE. In the UAE, CVD accounts for more than 25% of all deaths countrywide, however, this has increased in the major metropolitan center, accounting for 29% of all deaths in the Emirate of Abu Dhabi [6].

Diet, environmental factors, lifestyle, physical inactivity and genetics have been shown to contribute to the risk of NCDs [1]. Control of these primary risk factors could reduce the incidence of some NCDs by up to 80% and cancers by 40% [1]. As such, in recent years, there has been a significant effort to improve diet and increase physical activity to control the prevalence of NCDs. Hypertension is the most common outcome of excessive sodium intake independent of age and is a key risk factor for many NCDs [7]. Globally, over 7.5 million people die from hypertension-related complications per year, which surpasses deaths from tobacco smoking (5 million), obesity (2.8 million) and cholesterol (2.6 million) [8]. Hypertension, secondary to excessive salt consumption, is a major risk factor for CVD, responsible for 62% of strokes and 49% of coronary heart disease (CHD) [7]. In the UAE, approximately 30% of the population is hypertensive, [9] and the disease is thought to be widely underdiagnosed [10]. High salt intake is considered one of the major contributors to premature adult death in developed and developing countries [11]. A systematic review and meta-analysis of 5508 participants across 61 studies showed that higher salt intake was associated with significantly increased risk of stroke, stroke mortality and coronary heart disease [11]. Unfavorably high sodium intake remains prevalent worldwide and varies widely, ranging from 4–17 g/day, with mainland China having the greatest intake, and some less developed island nations the lowest [12], but for many countries it remains well above the World Health Organization (WHO) recommendation of less than 5 g of salt intake per day [13]. The UK Food Standards Agency highlighted in 2012 that 75% of salt intake comes from processed foods and proposed that a reduction in the sodium content of processed food and drink would be required to achieve the recommended daily intake in the community [14,15].

Alongside appropriate sodium consumption, ensuring adequate intake of potassium is vital to ensuring normal mineral homeostasis and healthy blood pressure. Potassium is the paired ion for sodium in a range of different physiologies—from nerve transmission to renal function. Having adequate potassium in the diet ensures that the kidney is able to remove sodium from the plasma, and hence allows more effective regulation of blood pressure. While frank potassium deficiency (hypokalemia) is well understood and monitored, chronic, insufficient dietary intake is not, despite being associated with an increase in systolic blood pressure, and in the face of declining diet quality, insufficient intake is becoming more common [16]. Despite its important role in health, potassium intake has not been investigated in the UAE, however, globally, it has been reported that there is widespread dietary insufficiency, and this is likely to be mirrored in the Gulf region.

The impact of diet on NCDs is critical, and dietary approaches such as the Mediterranean diet have been suggested to have a role in improving health outcomes globally. The Mediterranean diet, with its high fruit and vegetable and low meat and processed food content, is an effective way to reduce salt and sodium intake, leading to a subsequent improvement in health outcomes [17]. Its high vegetable content also lends itself to improving potassium intake—making it an effective intervention to reduce hypertension [17].

There are numerous methods used for assessing salt intake, including estimation by weighing ingested food, dietary recall questionnaires, estimating the salt content of food before ingestion and taking measurements of 24-h (hr) sodium excretion [18]. Measurement of 24-h urinary sodium excretion is considered to be the golden standard for estimating daily sodium intake on the premise that the majority (90–95%) of sodium ingested is excreted via the urine [18].

The aim of this study was to assess sodium intake using 24-h urinary sodium excretion from a sample of the healthy UAE population and to assess their knowledge, attitudes and practices (KAP) surrounding salt intake.

## 2. Materials and Methods

### 2.1. Study Design and Participants

A cross-sectional study with an anonymized self-reported questionnaire and the collection of 24-h urine for the assessment of sodium, potassium and creatinine excretion was conducted between March 2015 and June 2015 in the UAE. The questionnaire consisted of items to assess participants’ attitudes, behavior and knowledge (KAP) regarding salt consumption and knowledge. The sample size was calculated based on the following formula to be representative of the UAE, with a confidence interval level of 95%.

Sample size of an unknown population was calculated by Cochrane’s formula (n = z^2^ × *p* × (1 − *p*)/e2), with z = level of confidence (for a level of confidence of 95%, z = 1.96); *p* = the estimated proportion of the population that presents the characteristic of having high knowledge *p* = 0.5; e = margin of error accepted, e = 0.05; N (sample size) = 385 participants, plus 20% estimated dropouts = approximately 461 participants. Hence, a sample of 530 healthy individuals were recruited to participate in the study from the seven Emirates (Abu Dhabi, Dubai, Sharjah, Ajman, Ras Al Khaimah, Fujairah and Umm Al Quwain) aged between 20 and 65 years. Two methods were used for recruitment: face to face recruitment at community, school or university events and posters displayed in shopping malls, health centers, schools and university hostels. The WHO/PAHO (2010) protocol for 24-h urine collection and analysis was used. Four age groups were considered for recruitment in the current study; 20–30, 31–40, 41–50 and 51–65 years old with a ratio of 1:1 male to female. This demographic was used to ensure a sample of the ‘healthy’ population—participants older than 65 are likely to have comorbid disease which may have affected the urine analysis. All participants provided written informed consent to participate in the study.

**Figure 1:**
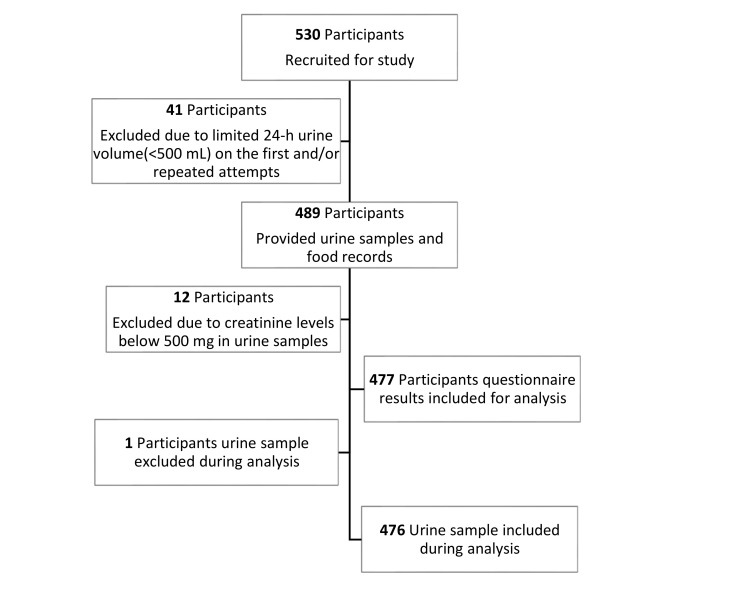
Flow diagram of the study design.

A screening questionnaire was designed to collect data regarding demographic information, lifestyle habits, past medical history, medication and current health status. Questionnaires were administered by the research team. Exclusion criteria at screening were those with self-reported chronic diseases (i.e., heart disease, using medication for hypertension, renal failure, liver disease), pregnant and lactating women, those on diuretics and women who had their menstrual period during the time of urine collection. Inclusion and exclusion criteria are summarized in Table 1. Inclusion criteria at screening were participants aged 20 to 65 years for both genders, non-pregnant and non-lactating, no known chronic kidney disease, renal failure, hypertension with medications and liver diseases, no medical condition(s) or medication(s) known to affect urination and able to collect 24-h (hr) urine. Exclusion criteria following urine collection included those that were unable to collect adequate urine within the 24-h time period (i.e., volume < 500 mL), and creatinine levels below 500 or above 2000 mg/day, which is equivalent to <9 or >26 mg/kg of body mass for female participants and <13 or >29 mg/kg of body mass for male participants [19]. Forty-one participants were excluded due to limited urine sample collection (<500 mL urine) or being unable to effectively urinate into the collection bottle, and 12 participants due to creatinine levels below 500 mg. The creatinine cutoffs were used to screen for renal abnormalities that may have skewed the results [19]. One urine sample was also excluded during testing due to abnormalities, leaving 476 urine samples for the final urine analysis, alongside 477 questionnaire responses. The enrolment process of the study participants is shown in Figure 1.

Participants were given full details of the study protocol with the opportunity to ask questions after which written informed consent to participate was sought. Each participant was allocated a personal identification number to provide anonymity and data confidentiality. Ethical approval for the study protocol was obtained from the UAE University (UAEU) Scientific Research Ethics Committee (Reference number: DVCRGS/36/2015).

### 2.2. Anthropometric Measurements

Body weight and height were measured for each participant and their body mass index (BMI) was calculated as weight (kg) divided by height (m) squared (kg/m^2^). Height was recorded to the nearest 1 cm using a stadiometer (Seca Stadiometer, Seca Ltd., Birmingham, UK) and weight was recorded using a balance (Biospace Co., Seoul, Korea) to the nearest 0.1 kg having removed their shoes and heaviest clothing. An appropriately trained member of the research team took all the measurements [20].

### 2.3. Knowledge, Attitude and Practice (KAP) Questionnaire

Participants were asked to complete a self-reported questionnaire. The questionnaire assessed knowledge relating to salt and health outcomes, frequency of consumption and their perceived salt consumption, and was developed according to the WHO/PAHO recommendations for the assessment of population sodium intake and behaviors. The development and performance of the specific questionnaire has been described elsewhere [21].

### 2.4. 24-h Urine Collections and Analysis

A single timed 24-h urine collection was obtained for the estimation of sodium excretion. Participants were given written and verbal instructions for the 24-h urine collection procedure. A 3-L coded plastic bottle was given to each participant for urine collection. Participants were asked to discard the first urine of the day and to collect all urine in the plastic bottle provided over the following 24-h. Participants were also asked to write on a separate sheet the time and date at the start and end of the urine collection, indicating occasions they missed urination. Urine samples with less than 500 mL or those who missed urine collection were rejected and participants were asked to repeat the process on another day.

Urine analysis for sodium, potassium and creatinine were conducted in the College of Food and Agriculture laboratories at UAEU. For the measurement of sodium and potassium levels in the urine, 50 mL of the urine sample was mixed with 200 µL of 1% nitric acid. Analytical solutions were introduced to a Varian ICP-OES model 710-ES spectrometer for sodium and potassium measurements [18]. Urinary creatinine was measured using a Cary 50 MPR Micro plate Reader-Varian and the concentration determined using a standard curve [22].

### 2.5. Statistical Analysis

Continuous variables were summarized by means and standard deviations or medians and inter-quartile ranges (25th–75th percentile) as appropriate. Continuous variables were checked visually for any departure from normality using histograms and quantile-quantile plots (Q–Q plots). All continuous data were reasonably normally distributed and therefore no transformations were applied. Categorical variables from the KAP questionnaire were reported as the percentage of responses per category. The Student’s *t*-test was used to compare the mean difference in sodium and potassium excretion in urine against the recommended dietary allowance. Measures of association for categorical variables were evaluated using a chi-square or Fisher’s exact test as appropriate. All analyses were conducted using the Statistical Package for the Social Sciences (SPSS) version 21. All statistical significance was determined at 5%.

## 3. Results

### 3.1. Characteristics of the Study Population

A total of 530 participants provided urine specimens, out of which 41 participants were excluded due to limited urine sample collection (<500 mL urine), and 12 participants due to creatinine levels below 500 mg in the urine, to give a final sample size of 477. One urine sample was excluded from the sample during analysis, due to excessive creatinine levels, leading to one less participant in the urine analysis (n = 476). The mean age was 37.31 years (standard deviation (SD) = 12.5 years, range 20–65 years), of which 55% were female (Table 2). The mean weight, height and BMI for participants were 73.37 ± 15.4 kg, 165.8 ± 8.95 cm and 26.7 ± 5.15 kg/m^2^, respectively (Table 2). The prevalence of underweight, normal weight, overweight and obese individuals was 3.14%, 37.11%, 36.06% and 23.69%, respectively.

### 3.2. Major Findings of the Knowledge, Attitude and Practice (KAP) Questionnaire

The knowledge, attitude and practice (KAP) questionnaire (Table 3) indicated that the majority of the participants added salt during cooking (N = 393; 82.4%) and while eating (N = 315; 66%). Most participants reported that they always or sometimes use stock cubes during cooking (N = 346; 72.6%), and 69.1% reported that they were aware that high salt intake could cause serious health problems. However, a large proportion (62.1%) thought that their salt consumption was within the recommended amounts, with 60% claiming to have tried to control their salt or sodium intake. Most of the participants (45.2%) reported that high salt intake was associated with high blood pressure, followed by kidney stones (18.7%) and obesity (17.8%), but only 11.7% associated it with heart disease, and 6.5% with T2D. More than 75% of the participants reported that they considered processed foods as a high source of salt.

### 3.3. High Levels of Sodium Secretion in the UAE Population within 24-h Urine Collection

The mean 24-h urine volume was 1338.3 ± 553 mL, with a range of 550–4000 mL. The mean sodium excretion in urine was 2713.4 ± 713 mg. The average values for sodium excretion in urine exceeded the WHO recommendations of sodium intake of less than 2300 mg (Table 4) [23]. Of the 476 participants, 320 (67.4%) had a sodium excretion above the WHO recommended level of 2300 mg. Males were more likely (51.6%) to exceed the WHO recommendation compared to females (odds ratio (OR): 2.60; 95% confidence interval (CI): 1.71 to 3.96; *p* < 0.001). However, there were no significant differences by age of those surpassing the recommendation (OR: 0.99; 95% CI: 0.98 to 1.02; *p* = 0.98).

**Figure 2:**
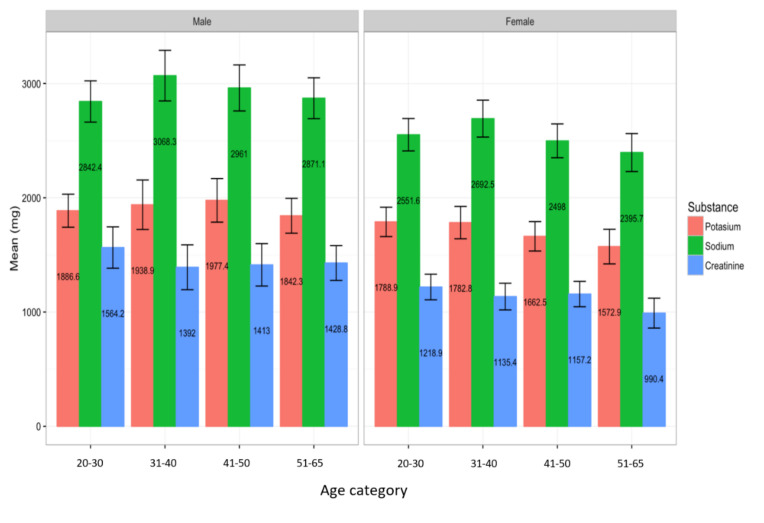
Mean creatinine, sodium and potassium levels for male (left panel) and female (right panel) participants according to age groups. The capped bars represent the 95% confidence intervals of the means.

Mean urinary excretion for potassium and creatinine and the sodium to potassium ratio were 1803.30 ± 618.03 mg, 1284.81 ± 607.0 mg and 1.64 ± 0.55 mg, respectively (Table 4). While it is challenging to use potassium excretion to estimate intake, it is likely that it is well below the WHO recommendations of 3500 mg/day [24]. Moreover, mean urinary excretion for creatinine for female participants was 13.42 ± 1.95 mg/kg body mass, with a minimum to maximum reading of 10.23 to 19.87 mg/kg body mass, while for male participants it was 21.81 ± 3.80 mg/kg body mass, with a minimum to maximum reading of 13.60 to 28.63 mg/kg body mass (Table 4). Mean urinary excretions for sodium, potassium and creatinine for male and female participants according to the different age groups are shown in Figure 2.

## 4. Discussion

This study showed that sodium intake of the participants exceeds WHO recommendations with concurrent low intakes of potassium. Moreover, most of the participants were unaware that their consumption was beyond the recommended levels of the WHO, however, most were able to identify some common sources of sodium, such as stock cubes and processed foods, as well as its deleterious effect on health. To our knowledge, this is the first study in the UAE reporting 24-h urinary sodium excretion. A 24-h collection period is necessary to capture the marked diurnal variation in sodium, chloride and water excretion. Electrolyte excretion in healthy individuals normally reaches the maximum at or before midday, and the minimum at night towards the end of sleep [25]. This study is also the first to report on potassium excretion in the UAE, another critical indicator of dietary hypertension risk.

The results from the current study were similar to the results in a study conducted in Eastern Saudi Arabia that showed the mean intake of sodium assessed by 24-h sodium excretion to be 3200 ± 1100 mg/day and 2700 ± 850 mg/day for men and women, respectively [26]. Similar findings were noted in a Jordan study using 24-h urinary sodium excretion, which showed that the average sodium intake was 4100 mg/day (10.4 g/day salt) and sodium intake was higher in males, 4300 mg, compared with 4000 mg by females. It was clear that the Jordanian participants consumed at least double the current WHO recommended daily sodium amount of 2000 mg (5 g salt) [27]. Likewise, a study conducted in Oman using the National Nutrition Survey based on a 24-h dietary recall noted the average intake of salt to be 11–12 g/day [28], again significantly higher than the WHO recommendation. Two further studies analyzing food consumption in Kuwait [29,30] reported the average salt intake to be within 8–10 g/day. These results, and our own, are strong indicators that consumption of sodium exceeds the WHO recommendations in the GCC countries. It is well known high sodium intake is associated with hypertension and stroke, as well as contributes to myocardial infarction and heart and kidney failure [31,32]. Consequently, this prevalent increase in sodium consumption is likely to contribute to the incidence of NCDs in the UAE. Globally, the mean intake of sodium is high in East Asia, Central Asia, Eastern Europe, Central Europe and the Middle East/North Africa, in the range of 3900–4200 mg/day, which is equivalent to 9.75–10.5 g/day of salt [33], far exceeding the WHO recommendations, and is similar to our findings. While higher than the recommendations, the results of our study would suggest that the UAE was at the lower end of the scale of sodium intake in these geographic areas, however, comparisons between urinary excretion and sodium intake must be drawn with care. This may reflect the relatively high levels of education and other key socio-economic indicators when compared to these countries, which may manifest in more health-promoting behaviors. Urinary excretion of sodium as a function of intake has also been assessed in other nations, with generally higher socio-economic and health indicators. For example, in Japan and the United Kingdom (UK), sodium intake was 4470 ± 1600 mg/day and 3289 mg/day, respectively, and was attributed to a high intake of canned and processed foods [34,35].

High dietary sodium and low dietary potassium intakes are associated with hypertension and increased risk of cardiovascular disease (CVD) [36]. In the current study, the sodium to potassium ratio was 1.64 ± 0.55, suggesting that not only did sodium intake exceed WHO recommendations but insufficient dietary potassium was also prevalent. The amount of potassium excreted in 24-h urine is well correlated with dietary potassium intake [37]. A high urinary sodium–potassium ratio is an indicator of a need to reduce sodium and increase potassium intake [1,3]. The WHO has suggested that achieving guidelines for sodium and potassium intake would yield a sodium–potassium ratio close to 1.00 [23,24].

In our study, 67.4% of the participants exceeded the WHO recommendations for salt intake, with more males (51.6%) than females exceeding the recommendations. This finding is consistent with previous studies conducted in Kuwait, where males (74.7%) and females (50.9%) exceeded recommendations [29]. Similarly, a study conducted in Eastern Saudi Arabia also found that males tend to consume more sodium compared to females [26]. This finding is also consistent outside the GCC countries. In Brazil, 90% of the of study population exceeded WHO recommendations for salt intake with excess consumption again more common in males [38]. Another study aimed to estimate sodium intake in New York City, noting the mean sodium intake to be 3239 mg/day, with 81% of participants exceeding recommendations [39]. Brown et al. (2013), reported that sodium intake tends to be higher in men than women, based on 5693 participants recruited in 1984–1987 aged 20–59 years from 29 North American and European samples [31]; the findings again echoed those in our study. The sex differences in sodium excretion could have a number of causes, however, it is likely that increased appetite and calorie consumption is a major driver behind the variance. It is also possible that socio-cultural norms lead men to make more salt-heavy diet choices in both social and home situations, both in the UAE and globally. This is particularly relevant when viewed against the increased risk profile of a number of NCDs in men, and also makes it more challenging for males to meet sodium guidelines, as they are required to reduce their intake considerably compared to women.

The data on urinary potassium excretion is also of significant importance to the health of the population of the UAE. It is well known that potassium is a key part of effective blood pressure regulation, because of its role in effective sodium clearance. The mean potassium intake shown in this study was well below the WHO guidelines, providing opportunities for improvement of the health of the UAE. Encouraging a varied diet, high in fruit and leafy vegetables, would provide a ready means of improving health outcomes. Potassium is not amenable to fortification, due to the negative consequences of excessive intake and a flavor-masking effect of common chemical formats of the mineral, which means improving diet quality is the major means of increasing intake in the community.

It was also found that 82.5% of the participants in this study added salt sometimes or always during cooking, which is similar to that noted in Lebanon, where 100% of the participants have been found to add salt during cooking [40]. In the current study, the majority of participants reported that they added stock cubes and additional table salt while eating, sometimes or always. These findings are similar to the Lebanon study which showed that 60% of the participants used table salt [40]. Likewise, 61% of university students in the UAE reported adding salt while cooking, and 14% of the participants often added salt to food even before tasting it [21].

Despite the fact that 67.4% of the participants exceeded the WHO recommended salt intake in the current study, only 20.0% reported that their consumption was beyond the recommended threshold (Table 3). In light of our data showing widespread sodium excess, this suggests that many people are unaware of how much they are consuming. In this regard, education and public awareness programs are required so that the general population is more aware of salt portion sizes and the sodium content of processed foods, drinks and other foods in general.

Interestingly, about 60% of the participants claimed to be taking measures to control their salt intake, again similar to reports from Lebanon (65.8%) [40]. These findings are also similar to a study conducted in five sentinel countries of the Americas (Argentina, Canada, Chile, Costa Rica and Ecuador), where almost 90% of the participants reported excess intake of salt is associated with adverse health conditions, and over 60% of the participants indicated they were conscious of their salt intake and taking measures to reduce it. They also found that more than 30% of their participants believed that reducing dietary salt intake was highly important [41]. Most of these studies reported that the majority of the participants were aware that high salt intake was associated with adverse health outcomes, however, this awareness does not translate into effective behavioral change in salt reduction. These interesting findings have some important implications for strategies to reduce sodium intake. One of the mainstays of sodium reduction is public education, however, these results would indicate that the general knowledge is adequate. However, education campaigns on effective ways to reduce intake, while increasing potassium and hydration, would play a role in the general reduction in sodium across the UAE, alongside effective regulations and sodium targets.

The results of the current study indicate that there is prevalent high sodium and low potassium intake within the general population of the UAE, which consequently may increase the risk of hypertension, CVD and other NCDs. This emphasizes the need for coordinated salt reduction programs to aid in the reduction of NCDs in the UAE. Strategies such as educational campaigns, regulation of sodium content of widely consumed food items and setting targets for sodium intake will allow for a more cohesive approach to improving the health of the UAE in this regard. In the last two decades, a number of countries have put sodium reduction strategies in place, and they have generally been somewhat successful, however, there are still significant strides to be made [42]. The most effective population interventions are likely to be salt reduction targets in common food stuffs, specific to the geographical areas and the local cuisines. In the metropolitan centers of the UAE, this will likely need to target processed and fast foods, which are becoming more of a staple in the Emirati diet, however, future research to identify significant sources of sodium in the UAE is needed to guide policy makers. Other nations with successful sodium reduction strategies have used salt targets, typically between 5–8 g/day [42], to help guide these regulations and interventions. Decreasing the sodium consumption of the UAE will also require regulation of industry to offer more low-salt options, as well as improve standards on labeling and nutritional declaration on packaged foodstuffs.

The Mediterranean diet could play a role in combatting the widespread salt imbalance in the UAE. The Mediterranean diet is inherently low in sodium and high in potassium due to its high vegetable and low meat and processed food content. The Mediterranean diet is also accessible to the local region, as it contains a number of similarities to traditional food practices in the Arab nations, such as an emphasis on vegetables, dairy, grains and spices, however, the Emirati cuisine traditionally features meat products more strongly [43]. The Mediterranean diet has been shown to reduce hypertension [44], however, there is some debate surrounding the role of sodium in this. Some authors have found that the Mediterranean diet does not readily offer reductions in sodium [45], however, this may be due to variations in adherence and specific components of the diet, as well as the amount of other minerals, such as potassium, which are abundant in plant-rich diets.

Despite the significant findings, a limitation of this study was that urinary sodium was assessed by a single 24-h urine collection and this may not represent the average sodium intake in a person due to daily individual variability. However, a single urine measurement is considered a more accurate measure of sodium intake at a population level [18], though it may possibly be less accurate for individuals. There is also a potential that the recruited population may have been broadly healthier, as they were likely more health conscious, better educated and possibly of a higher socio-economic status. Future studies should account for key socio-economic indicators, such as years of education and household income in both their recruitment and analysis to ensure a representative sample of the population. This may have led to an underestimation of the UAE’s sodium intake found in this study. Additionally, while the KAP questionnaire used in this study captures some important facets of the participants’ knowledge and behavior surrounding salt, there is further room for additional information, particularly surrounding important sources of sodium in the modern diet. An expanded questionnaire, and other measures such as food diaries, would provide more reliable information on the true intake of sodium, and how that compares to the participants’ knowledge. Despite these limitations, the study described was statistically sound and powered to reliably identify the sodium practices in the UAE. The large sample, with demographically representative participants, and the validated analytical methods, are also strengths of the research presented here.

## 5. Conclusions

In this cross-sectional sample of the UAE population, the majority consumed salt well above the international WHO recommendations with a concurrent low intake of potassium, suggesting significant room for improvement in the intake of these minerals. There are significant differences by gender, with males more likely to exceed the WHO recommendations for salt and sodium intake.

It is imperative for communities, as well as local and national governments, to play a leading role in the development and implementation of salt reduction strategies, such as increasing adherence to the Mediterranean diet, as well as setting standards for industry. Future research should aim to validate these findings with larger studies, as well as to identify important sources of sodium in the diet of the UAE. Awareness programs should be established to educate the population about the risk factors for excess salt consumption. A nationwide assessment should be conducted to evaluate the level of the problem, and to enable identification of appropriate priorities for the implementation of population-based diet-related interventions to reduce the prevalence of NCDs and their associated rates of morbidity and mortality.

## Figures and Tables

**Table 1:** Summary of inclusion and exclusion criteria.

Inclusion	Exclusion
Age 20–65	Renal or urinary pathology
Non-pregnant	Chronic disease
Non-lactating	Current menstrual period
No chronic kidney disease	24-h urine volume <500 mL
No medical conditions	Urine creatinine <500 mg or >2000 mg
Not currently taking prescribed medications known to affect urine	

**Table 2:** Summary of study population demographics (N = 477).

Variable	Mean ± SD
Age (years)	37.31 ± 12.5
Weight (kg)	73.37 ± 15.4
Height (cm)	165.8 ± 8.95
Body Mass Index (BMI)	26.7 ± 5.15
	N (%)
Emirates ^1^	
Abu Dhabi (West)	137 (28.72)
Al-Ain (East)	150 (31.45)
Northern Emirates ^2^	190 (39.83)
Age Category (years)	
20–30	156 (32.70)
31–40	113 (23.69)
41–50	123 (25.78)
51–65	85 (17.82)
BMI Classifications (WHO definition)	
Underweight (<18.5 kg/m^2^)	15 (3.14)
Normal-weight (18.5–24.9 kg/m^2^)	177 (37.11)
Overweight (25.0–29.9 kg/m^2^)	172 (36.06)
Obese (30.0–34.9 kg/m^2^)	113 (23.69)
Gender Distribution, N (%)	
Males	214 (44.86)
Females	263 (55.14)

^1^ Geographic divisions of United Arab Emirates; ^2^ Dubai, Sharjah, Ajman, Umm Al Quwain and Fujairah.

**Table 3:** Knowledge, attitude and practice (KAP) of salt intake and participants’ knowledge on health consequences (N = 477). * This question is multiple choice with more than one selection allowed.

	Gender			Chi-Square (*p*-Value)	Age Category (Year)				Chi-Square (*p*-Value)
Do you Add Salt during Cooking (Missing Answers = 0)	Male N = 214 (%)	Female N = 263 (%)	Total N = 477 (%)		20–30 N = 156 (%)	31–40 N = 113 (%)	41–50 N = 123 (%)	51–65 N = 85 (%)	
Never	46 (21.6)	38 (14.1)	84 (17.5)	5.60 (0.061)	28 (18.0)	26 (23.0)	13 (10.6)	17 (19.0)	20.01 (0.01)
Sometimes	80 (37.6)	96 (36.6)	176 (37.1)		65 (41.7)	42 (37.2)	46 (37.4)	23 (27.4)	
Always	88 (40.8)	129 (49.2)	217 (45.5)		63 (40.4)	45 (39.8)	64 (52.0)	45 (54.2)	
**Do you Add Salt to Food at the Table (Missing Answers = 1)**	**Male** **N (%)**	**Female** **N (%)**	**Total** **N = 476 N (%)**	**Chi-Square (*p*-Value)**	**20–30** **N (%)**	**31–40** **N (%)**	**41–50** **N (%)**	**51–65** **N (%)**	**Chi-Square (*p*-Value)**
Never	71 (33.3)	90 (34.4)	161 (33.8)	2.12 (0.548)	54 (34.6)	31 (27.4)	40 (32.8)	36 (42.8)	12.71 (0.391)
Sometimes	93 (43.7)	122 (46.60	215 (45.2)		75 (48.1)	53 (46.9)	51 (41.8)	36 (42.8)	
Always	49 (23.0)	51 (19.0)	100 (21.0)		27 (17.3)	29 (25.7)	31 (25.4)	13 (14.3)	
**Do you Use Stock Cubes during Cooking (Missing Answers = 0)**	**Male** **N (%)**	**Female** **N (%)**	**Total** **N = 477 N (%)**	**Chi-Square (*p*-Value)**	**20–30** **N (%)**	**31–40** **N (%)**	**41–50** **N (%)**	**51–65** **N (%)**	**Chi-Square (*p*-Value)**
Never	55 (25.8)	76 (28.6)	131 (27.4)	0.50 (0.779)	39 (25.0)	30 (26.6)	33 (26.8)	29 (34.1)	4.65 (0.794)
Sometimes	61 (28.6)	74 (28.2)	135 (28.4)		42 (26.9)	35 (31.0)	36 (29.2)	22 (25.9)	
Always	98 (45.5)	113 (43.1)	211 (44.2)		75 (48.1)	48 (42.5)	54 (44.0)	34 (40.0)	
**How Much Salt do you Think you Consume (Missing Answers = 0)**	**Male** **N (%)**	**Female** **N (%)**	**Total** **N = 477 N (%)**	**Chi-Square (*p*-Value)**	**20–30** **N (%)**	**31–40** **N (%)**	**41–50** **N (%)**	**51–65** **N (%)**	**Chi-Square (*p*-Value)**
Too much	49 (23.0)	47 (17.6)	96 (20.0)	2.85 (0.240)	32 (20.5)	26 (23.0)	22 (18.0)	16 (19.1)	3.101 (0.928)
Just the right amount	125 (58.2)	171 (65.3)	296 (62.1)		96 (61.5)	70 (62.0)	80 (65.0)	50 (59.5)	
Far too little	40 (18.8)	45 (17.2)	85 (17.9)		28 (17.9)	17 (15.0)	21 (17.0)	19 (21.4)	
**Do you Think that High Salt Diet could Cause Serious Health Problems? (Missing Answers = 0)**	**Male** **N (%)**	**Female** **N (%)**	**Total** **N = 477** N (%)	**Chi-Square (*p*-Value)**	**20–30** **N (%)**	**31–40** **N (%)**	**41–50** **N (%)**	**51–65** **N (%)**	**Chi-Square (*p*-Value)**
Yes	149 (69.6)	181 (68.8)	330 (69.1)	3.682 (0.158)	113 (72.4)	73 (64.6)	84 (68.4)	60 (70.0)	4.782 (0.780)
No	45 (21.0)	68 (25.8)	113 (23.7)		32 (20.6)	32 (28.3)	31 (25.2)	18 (23.7)	
Don’t know	20 (9.3)	14 (5.4)	34 (7.2)		11 (7.1)	8 (7.1)	8 (6.56)	7 (8.4)	
What are the Health Problems Associated with High Salt Intake *	**Male** **N (%)**	**Female** **N (%)**	**Total** **N = 477N (%)**	**Chi-Square (*p*-Value)**	**20–30** **N (%)**	**31–40** **N (%)**	**41–50** **N (%)**	**51–65** **N (%)**	**Chi-Square (*p*-Value)**
High blood pressure	90 (42.1)	126 (47.9)	216 (45.2)	3.43 (0.414)	73 (46.8)	47 (41.6)	59 (48.0)	37 (43.5)	4.23 (0.624)
Kidney stones	43 (20.1)	46 (17.5)	89 (18.7)		31 (19.9)	22 (19.5)	21 (17.1)	15 (17.6)	
Obesity	38 (17.8)	47 (17.9)	85 (17.8)		25 (16.0)	23 (20.3)	18 (14.6)	19 (22.3)	
Diabetes	18 (8.4)	13 (4.9)	31 (6.5)		8 (5.1)	9 (8.0)	8 (6.5)	6 (7.1)	
Heart disease	25 (11.7)	31 (11.8)	56 (11.7)		19 (12.2)	12 (10.6)	17 (13.8)	8 (9.4)	
**Do you Think Processed Foods are High in Sodium? (Missing Answers = 0)**	**Male** **N (%)**	**Female** **N (%)**	**Total** **N = 477 (%)**	**Chi-Square (*p*-Value)**	**20–30** **N (%)**	**31–40** **N (%)**	**41–50** **N (%)**	**51–65** **N (%)**	**Chi-Square (*p*-Value)**
Yes	159 (74.2)	207 (78.6)	366 (76.7)	1.620 (0.444)	123 (78.9)	88 (77.9)	88 (71.5)	68 (80.0)	6.578 (0.574)
No	55 (25.8)	56 (21.4)	111 (23.3)		33 (21.1)	25 (22.1)	35 (28.5)	17 (20.0)	
**Do you do Anything on a Regular Basis to Control your Salt or Sodium Intake? (Missing Answers = 0)**	**Male** **N (%)**	**Female** **N (%)**	**Total** **N = 477 (%)**	**Chi-Square (*p*-Value)**	**20–30** **N (%)**	**31–40** **N (%)**	**41–50** **N (%)**	**51–65** **N (%)**	**Chi-Square (*p*-Value)**
Yes	131 (61.2)	158 (60.1)	289 (60.6)	0.935 (0.626)	88 (56.4)	69 (61.1)	76 (61.8)	56 (65.9)	4.823 (0.764)
No	83 (38.8)	105 (39.9)	188 (39.4)		68 (43.6)	44 (38.9)	47 (38.2)	29 (34.1)	

**Table 4:** Mean sodium, potassium and creatinine urinary excretion and ratio of sodium to potassium (n = 476).

Nutrients	Mean ± SD	Recommendation	*p*-Value
Mean 24-h sodium excretion in urine (mg)	2713.40 ± 713	<2300 mg	<0.001
Mean 24-h potassium excretion in urine (mg)	1803.30 ± 618.03	>3510 mg	<0.001
Mean 24-h creatinine excretion in urine (mg)	1284.81 ± 607.0		
Mean 24-h creatinine (mg/kg body mass)	16.83 ± 4.84		
Mean 24-h creatinine (mg/kg body mass)—female	13.42 ± 1.95		
Mean 24-h creatinine (mg/kg body mass)—male	21.81 ± 3.80		
Mean 24-h urinary Na/K ratio	1.64 ± 0.55		
